# Synthesis of Peptides from **α**- and **β**-Tubulin Containing Glutamic Acid Side-Chain Linked Oligo-Glu with Defined Length

**DOI:** 10.1155/2010/189396

**Published:** 2010-12-15

**Authors:** Werner Tegge, Carlos F. S. Bonafe, Aileen Teichmann, Christian Erck

**Affiliations:** ^1^Department of Chemical Biology, Helmholtz Centre for Infection Research, Inhoffenstraße 7, 38124 Braunschweig, Germany; ^2^Departamento de Bioquimica, Universidade Estadual de Campinas, Sao Paulo 13085-135, Brazil; ^3^Synaptic Systems, Inhoffenstraße 7, 38124 Braunschweig, Germany

## Abstract

Side-chain oligo- and polyglutamylation represents an important posttranslational modification in tubulin physiology. The particular number of glutamate units is related to specific regulatory functions. In this work, we present a method for the synthesis of building blocks for the Fmoc synthesis of peptides containing main chain glutamic acid residues that carry side-chain branching with oligo-glutamic acid. The two model peptide sequences CYEEVGVDSVEGEG-E(E_*x*_)-EEGEEY and CQDATADEQG-E(E_*x*_)-FEEEEGEDEA from the C-termini of mammalian **α**1- and **β**1-tubulin, respectively, containing oligo-glutamic acid side-chain branching with lengths of 1 to 5 amino acids were assembled in good yield and purity. The products may lead to the generation of specific antibodies which should be important tools for a more detailed investigation of polyglutamylation processes.

## 1. Introduction

Enzymatic side-chain oligoglutamylation of tubulin was first reported in 1990 [1]. It is an important posttranslational modification for the regulation of cellular functions like mitosis and axonal growth [2, 3]. This process has been termed side-chain “polyglutamylation,” which is somewhat misleading, since the number of side-chain glutamic acids is small in most cases and seems generally to be well defined. The length of the oligo-Glu side chain can vary between 1 and 20, and possibly even more residues and fine tuning of protein interactions seems to depend critically on the exact number of the Glu units. Although proteomics approaches have shown that more than 100 proteins are modified this way [4], as yet very little is know about this process. One of the obstacles in the investigations is the lack of specific antibodies that can discriminate between the different lengths of the oligo-Glu modifications. So far mainly the monoclonal antibody GT335 [5] has been used in studies of side-chain oligo- and polyglutamylation of proteins. The antibody was generated with a peptide containing a branching with two glutamic acids. The peptide had been obtained by chemical Boc synthesis with the coupling of benzyl-protected di-glutamic acid to a selectively deprotected Glu side chain by on-resin activation with N-hydroxy-succinimide and carbodiimide [6]. Although the antibody GT335 is valuable for the identification of side-chain glutamylation in general, it is insensitive to the length of the modification. For many investigations in this field, the availability antibodies that are specific to the length of the side chain would be highly advantageous. 

For the generation of such antibodies, the synthesis of peptides with corresponding defined side chains is mandatory. We have investigated two different strategies for the chemical Fmoc synthesis of peptides containing oligo-Glu branching. Firstly the incorporation of the main chain glutamic acid with a selectively cleavable allyl side-chain protection, followed by selective deprotection, activation of the carboxylic group on the resin, and the coupling of H-Glu(tBu)-Oallyl with further side-chain elongation after allyl cleavage, similar to the Boc method published before. Secondly the preassembling of suitably protected building blocks with the required side-chain length and their utilization in the assembly of the main chain.

**Figure 1:**
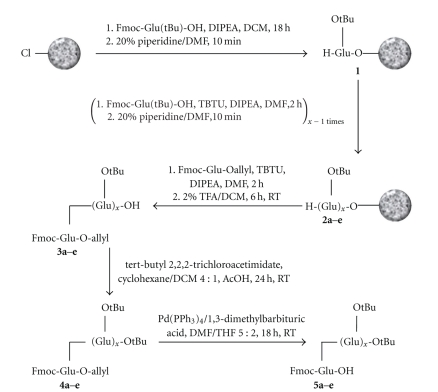
General reaction scheme for the synthesis of the Fmoc-protected building units for solid-phase peptide synthesis (*x* = 1 to 5, corresponding to the incrementations **a** to **e** of the numbering of the compounds).

The first strategy turned out to be not suitable, since according to HPLC and MALDI analysis only one or two side-chain Glu residues could be introduced, and even that is not reliable (data not shown). The second procedure proved to be much more useful, and the desired sequences were obtained in good yield and purity. This strategy is described in detail below (Figure 1).

Synthesis of the building blocks was carried out by first immobilizing Fmoc-Glu(tBu)-OH on chlorotrityl chloride resin (**1**), followed by elongation with the required number of the same building block, and finally coupling of Fmoc-Glu-Oallyl via its side-chain carboxyl function (**2a–e**). After cleaving the protected peptides from the support by the use of mild acid (2% trifluoroacetic acid in dichloro methane), the resulting free carboxylic function of **3a–e** was esterified by treatment with t-butyl 2,2,2-trichloroacetimidate by a modification of the method by Armstrong et al. [7]. The authors have used the Lewis acid boron trifluoride etherate as a catalyst. Although in other investigations Fmoc amino acids appeared stable towards boron trifluoride etherate [8], we experienced loss of compounds **3a–e** with no product formation under those conditions. Introduction of the t-butyl group proceeded smoothly without boron trifluoride etherate, though, after treatment with t-butyl 2,2,2-trichloroacetimidate over an extended time period in the presence of acetic acid. The side reaction of esterification of the acetic acid with the reagent t-butyl 2,2,2-trichloroacetimidate was counterballanced by the use of a tenfold excess of the t-Bu transfer reagent over the amount of peptide. In the presence of acetic acid, the reaction smoothly went to completion to yield the compounds **4a–e** within one to two days. Finally cleavage of the allyl function with the palladium catalyst tetrakis(triphenylphospine)palladium(0) and 1,3-dimethylbarbituric acid resulted in the desired products **5a–e** without indication of a loss of the Fmoc group.

**Figure 2:**
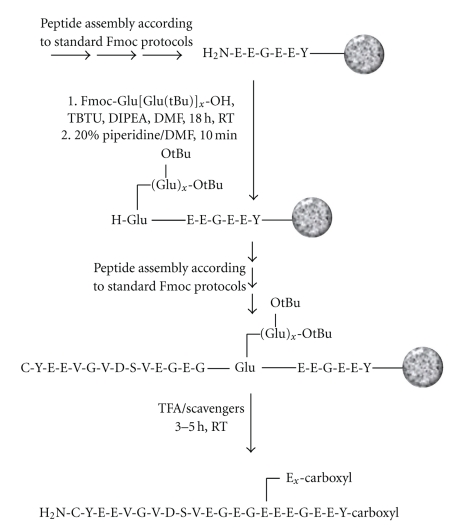
Scheme for the assembly of the carboxy-terminal sequence of *α*1-tubulin containing a side-chain oligo-glutamic acid branched glutamic acid at position 15 of the sequence. The corresponding *β*1-tubulin peptides (CQDATADEQG-E(E_*x*_)-FEEEEGEDEA) were assembled accordingly (*x* = 1 to 5, corresponding to the incrementations a to e of the numbering of the compounds).

Model peptides corresponding to the C-terminal sequences of mammalian *α*1-tubulin (CYEEVGVDSVEGEGEEEGEEY) and *β*2-tubulin (CQDATADEQGEFEEEEGEDEA) with an extra cysteine at the N-terminus for immobilization were assembled that contain oligo-Glu side chains of 1 to 5 residues at positions 15 and 11, respectively (underlined). Fmoc peptide synthesis protocols were employed (Figure 2), and the preassembled olig-Glu building blocks were used in twofold excess with coupling times of 18 hours. The products were obtained in good yield and purity and are currently used in the generation of specific antibodies. 

The procedure described in this study should facilitate the synthesis of different oligo-Glu side-chain modified peptides and should open new approaches that lead to a better understanding of the fundamental process of side-chain oligoglutamylation. 

## 2. Experiments

### 2.1. Resin Loading

Chloro-(2′-chloro)trityl polystyrene resin (capacity 1.09 mmol/g, Rapp Polymere, Tübingen, Germany) was treated with Fmoc-Glu(OtBu)-OH (anhydrous, 1.5 mmol/g resin) and DIPEA (6 mmol/g resin) in dry DCM for 18 h. After washing with DMF, the Fmoc group was cleaved with 20% piperidine in DMF, and the dibenzofulvene adduct was quantified photometrically [9]. A loading of 660 *μ*mol/g dry resin was determined.

### 2.2. Assembly of the Precursors 3a–e (General Procedure)

To the tritylchloride resin with attached Fmoc-Glu(OtBu) **1** (500 *μ*mole), the required numbers of Fmoc-Glu(OtBu)-OH units (0–4) were coupled in a 5-fold excess by activation with TBTU (1 equivalent) and DIPEA (2 equivalents) in DMF for 2 hours, in each case followed by a Fmoc cleavage with 20% piperidine in DMF over 10 min. Finally Fmoc-Glu-Oallyl was coupled with the same activation strategy. The solid supports with the attached products were treated with 100 ml of 2% TFA in DCM in portions of 5 ml over a period of 6 hours and the filtrate collected in 200 ml of DMF. After evaporation of the solvents, the residues were dissolved in 40 ml of dioxane and lyophlilized three times to give the products **3a–e** as white powders in yields of 95–98%. NMR (CD_2_Cl_2_) of 3a exemplarily for the group: 7.83–7.74 (2 H, d, Fmoc), 7.69–7.56 (2 H, d, Fmoc), 7.48–7.38 (2 H, t, Fmoc), 7.38–7.29 (2 H, t, Fmoc), 7.08–6.97 (1 H, d, NH), 6.04–5.85 (2 H, m, NH and –CH=C allyl), 5.40–5.20 (2 H, m (together with CHDCl_2_), =CH_2_ allyl), 4.70–4.59 (2 H, d, –CH_2_–allyl), 4.59–4.30 (4 H, m, 2*x* C*α* and –CH_2_–O Fmoc), 4.30–4.18 ( 1 H, t, CH Fmoc), 2.52–1.89 (8 H, m, 2*x*–CH_2_–CH_2_– Glu side chain), 1.50–1.40 (9 H, –CH_3_ tBu).

### 2.3. Formation of the t-Butyl Esters 4a–e (General Procedure)

The above products in amounts corresponding to 500 *μ*mole were dissolved in 10 ml of DCM, and 40 ml of cyclohexane was added. After the addition of tert-butyl 2,2,2-trichloroacetimidate (1.79 ml, 5 mmole) and acetic acid (572 *μ*l, 10 mmole), the mixtures were stirred for 24 h to 48 h at room temperature with occasional control by analytical HPLC (Phenomenex Gemini 5 *μ*m, 50 × 2 mm column with a gradient of 5% acetonitrile in water to 100% acetonitrile containing 0.1% TFA). After completion of the reaction, the organic layer was extracted three times with 0.1 N HCl and dried with magnesium sulfate. The solvents were removed, and the crude products were purified by HPLC on a 250 × 40 mm Nucleosil C18 100-7 column (Macherey-Nagel, Düren, Germany) with gradients from 50% methanol in water to 100% methanol at 25 ml/min in 1 h. The products **4a–e** were obtained in yields of 80-90%. NMR (CD_2_Cl_2_) of **4a** exemplarily for the group: 7.82–7.75 (2 H, d, Fmoc), 7.67–7.58 (2 H, d, Fmoc), 7.43–7.37 (2 H, t, Fmoc), 7.37–7.29 (2 H, t, Fmoc), 6.46–6.37 (1 H, d, NH), 5.99–5.86 (1 H, m, –CH=C allyl), 5.86-5.79 (1 H, d, NH), 5.38-5.21 (2 H, m (together with CHDCl_2_), =CH_2_ allyl), 4.68-4.58 (2 H, d, –CH_2_– allyl), 4.47–4.28 (4 H, m, 2*x* C*α* and –CH_2_–O Fmoc), 4.28–4.19 (1 H, t, CH Fmoc), 2.36–1.83 (8 H, m, 2*x* –CH_2_–CH_2_– Glu side chain), 1.52–1.33 (18 H, –CH_3_ tBu).

### 2.4. Cleavage of the Allyl Group

Amounts given for 100 *μ*mole peptide: Cleavage of the allyl group was carried out by dissolving tetrakis(triphenylphospine)palladium(0) (11.5 mg, 10 *μ*mole) and 1,3-dimethylbarbituric acid (15.6 mg, 100 *μ*mole) in an argon-saturated mixture of DMF (5 ml) and THF (2 ml). The solution was added to the peptide under argon atmosphere, and the mixture was kept for 18 h in the dark at room temperature. Completion of the cleavage of the allyl group was confirmed by analytical HPLC. The solvents were removed by evaporation and, the residues were taken up in ethyl acetate and extracted three times with 0.1 N HCl. The organic layer was dried with magnesium sulfate and evaporated to yield the crude products **5a–e**, which were purified by preparative HPLC on a 250 × 40 mm Nucleosil C18 100-7 column (Macherey-Nagel, Düren, Germany) with a gradient from 50% methanol in water to 100% methanol at 25 ml/min in 1 h. NMR analyses (CDCl_3_) showed the disappearance of the allyl signals at 4.60 ppm–4.68 ppm (2 H, d, O–CH_2_–), 5.20–5.38 (2 H, dd, C=CH_2_) and 5.81 ppm–5.98 ppm (1 H, m, –CH=C). The products had purities of 90% to 95% as judged by analytical HPLC. Product yields of 80%–90% were obtained. Mass analyses (Bruker Daltonics MALDI, calculations are reported for monoisotopic masses): 5a: calc. for C33H42N2O9: 610.29, found: 633.15 (M+Na)^+^; 5b: calc. for C42H57N3O12: 795.39, found: 818.33 (M+Na)^+^; 5c: calc. for C51H72N4O15: 980.50, found: 1003.40 (M+Na)^+^; 5d: calc. for C60H87N5O18: 1165.60, found: 1188.50 (M+Na)^+^; 5e: calc. for C79H102N6O21: 1350.71, found: 1373.65 (M+Na)^+^. 

### 2.5. Incorporation of the Oligo-Glu Building Blocks 5a–e into the Peptide Sequences

For the *α*1-tubulin series the peptide EEGEEY was synthesized on a 250 *μ*mole scale on TentaGel S PHB-Tyr(tBu)-Fmoc resin with a pioneer automated peptide synthesizer (Applied Biosystems, Foster City, USA) with TBTU/DIPEA for activation and a fourfold excess of amino acids. Coupling time was two hours. The resin was washed with DCM, dried, and divided into five equal portions of 50 *μ*mole. To each of these portions, two equivalents of the different building blocks **5a–e** were coupled by activation with TBTU (1 equivalent) and DIPEA (2 equivalents) in DMF over 18 h. After test cleavages of 5 mg of the resulting material (95% TFA, 3% triisopropylsilane, 2% water), which unequivocally showed complete incorporation of the building blocks, the remaining sequence (CYEEVGVDSVEGEG) was assembled on a Syro Multiple Peptide Synthesizer (MultiSyn Tech, Witten, Germany) by employing TBTU/DIPEA for activation and 1-hour coupling times. After washing the resin with DCM, the peptides were cleaved from the support and deprotected by a 4 h treatment with TFA/triisopropylsilane/water (95 : 3 : 2), precipitated with t-butylmethyl ether, and characterized by HPLC (Phenomenex Gemini 5 *μ*m, 50 × 2 mm column with a gradient of 95% water to 100% acetonitrile containing 0.1% TFA) and MALDI mass spectrometry.

The *β*2-tubulin peptides were generated accordingly by preassembling the sequence FEEEEGEDEA on TentaGel S PHB-Ala Fmoc, manually coupling the oligo-Glu building units **5a–e** in a twofold excess, and finally assembling the sequence CQDATADEQG on a Syro Multiple peptide synthesizer. All conditions for synthesis, workup, and analysis are as described above. All mass analyses obtained by MALDI corresponded to the expected values.
